# MicroRNA-126 contributes to Niaspan treatment induced vascular restoration after diabetic retinopathy

**DOI:** 10.1038/srep26909

**Published:** 2016-05-26

**Authors:** Yang Wang, Hua Yan

**Affiliations:** 1 Department of Ophthalmology, Tianjin Medical University General Hospital, No. 154, Anshan Road, Tianjin, 300052, China

## Abstract

Diabetic retinopathy (DR) is a serious microvascular complication of diabetes and a major cause of blindness in the developing world. Early diabetic retinopathy is characterized by a loss of pericytes and vascular endothelial cells, a breakdown of the blood–retinal barrier, vascular dysfunction and vascular-neuroinflammation. However, optimal treatment options and related mechanisms are still unclear. MicroRNA-126 (miR-126) plays a potential role in the pathogenesis in DR, which may regulate VEGF, Ang-1 and VCAM-1 expressions. This study investigated the therapeutic effects and mechanisms of Niaspan treatment of DR in diabetes (DM) rats. DM rats exhibits significantly decreased miR-126 and tight junction Claudin-5/Occludin/ZO-1 genes expression, and increased Blood retinal-barrier (BRB) breakdown, retinal apoptosis and VEGF/VEGFR, as well as VCAM-1/CD45 expressions in the retina compared to normal control group. Niaspan treatment significantly improved clinical and histopathological outcomes; decreased the expressions of VEGF/VEGFR, VCAM-1/CD45, apoptosis and BRB breakdown, significantly increased tight junction proteins and Ang-1/Tie-2 expressions, as well as increased retinal miR-126 expression compared to non-treatment diabetic rats. These data are the first to show that Niaspan treatment ameliorates DR through its repair vascular and inhibits inflammatory effects, and also suggest that the miR-126 pathway may contribute to Niaspan treatment induced benefit effects.

Diabetic retinopathy (DR) is a major cause of blindness in the developing world^1^. According to the World Health Organization report in 2015, that diabetes currently affects nearly 347 million people worldwide. DR is a serious microvascular complication of diabetes and a major cause of blindness which usually affects people ranging in age 30 to 70 years^2^. Diabetes affects the entire neurovascular unit of the retina, with gradual neurodegeneration, gliosis, neuroinflammation, vascular abnormalities including plasma leakage, compromise of the vascular blood-retinal barrier (BRB), edema, angiogenesis, and eventual fibrosis, occur at increasing frequency^3^. However, treatment options for DR remain limited and with adverse effects, therefore, diabetic patients undergo a very high risk of eventual blindness. Tight control of blood sugar levels is the only proven preventative measure^4^. For several decades, Laser photocoagulation remain as the conventional approach for treating vascular dysfunction in DR such as macular edema^5^, while this procedure preserves central vision at the expense of loss of peripheral vision. Vascular endothelial growth factor (VEGF) stimulates endothelial cell growth and causes vascular permeability. Targeting VEGF has proved highly effective in the procedure of proliferative diabetic retinopathy (PDR). However, it requires repeated treatment and may impair neuronal and vascular survival function^6^. Therefore, there is an emerging need to develop new therapeutic approaches for this devastating disease.

High-density lipoprotein (HDL) is associated with a decreased risk for coronary heart disease and has a positive effect on endothelial cell and vascular wall function^7^. Higher HDL level is also correlated with decreased prevalence of PDR^8^. Niacin (vitamin B3 or nicotinic acid) is the most effective medication in current clinical use for increasing HDL cholesterol and it substantially lowers total cholesterol and triglycerides^9^. Niaspan is a prolonged release formulation of niacin and is safely used in patients with diabetes^10^. Some studies have shown that Niaspan treatment increased Angiopoietin-1 (Ang-1), but attenuated Angiopoietin-2 (Ang-2) expression in type 1 diabetes rats^11^. Meantime, Niaspan treatment promotes vascular remodeling and improves functional outcome after stroke^12^. However, the therapeutic effects of Niaspan on the DR have yet to be elucidated.

MiRNAs are small noncoding RNAs on average only 22 nucleotides, that are thought to regulate gene expression through sequence-specific base pairing with target mRNAs^13^. It has been reported that endothelial cells of mature vessels express high levels of noncoding RNAs called microRNA-126 (miR-126), promotes angiogenesis in response to angiogenic growth factors^14^. Additional deletion of miR-126 in mice causes leaky vessels, hemorrhaging, due to a loss of vascular integrity^15^. Thus, targeting the expression of miR-126 may be a novel therapeutic approach for diseases involving excess or insufficient vasculature. Whether Niaspan regulates miR-126 in the diabetic retinopathy has not been investigated.

In this study, we examined the effects of Niaspan on streptozotocin induced DR. We demonstrate that Niaspan administered three months after diabetes induction significantly improves functional outcome, concomitantly with increasing vascular repair in the retina. The probable molecular bases for this vascular remodeling induced by Niaspan are described here.

## Research Design and Methods

### Animals

Adult Male Wistar rats (225–250 g) purchased from the Academy of Military Medical Science (Beijing, China), were housed in specific pathogen-free conditions with water and food available ad libitum. All procedures involving rat were approved by the Laboratory Animal Care and Use Committee of Tianjin Medical University, and also conformed to the Association for Research in Vision and Ophthalmology Statement for the Use of Animals in Ophthalmic and Vision Research.

### Diabetes induction and treatment

Diabetes was induced by injection of streptozotocin (STZ, 45 mg/kg, Sigma Chemical Co., USA) via tail vein in Wistar rats. One week after STZ injection, rats exhibiting hyperglycemia (blood glucose ≥ 16.7 mmol/L) were considered diabetic and were subjected to outline experiments^16^. Niaspan (Resources Saike pharmaceutical Co., China) was given 40 mg/kg/day dissolved in water after injection of STZ (day-7). Ninety rats were divided into: 1) Normal control (CON) group (30 rats); 2) Diabetic retinopathy (DR) model without Niaspan treatment group (30 rats); 3) DR rats treated with Niaspan (NA) group (30 rats).

### Cell culture and treatment with miRNA inhibitor

Human retinal endothelial cells (HREC) were purchased from ScienCell Research Laboratories (America). All cells were grown in Endothelial Cell Medium (ECM) (ScienCell, America). Cells were treated for 72 h with ECM containing of the following: 5 mM D-glucose (euglycemic control)(CON group); 25 mM D-glucose (hyperglycemia) (HI group); 25 mM D-glucose (hyperglycemia + Niacin (1 mM, Sigma Chemical Co., USA) (NA group); 25 mM D-glucose (hyperglycemia) + Niacin (1 mM) + microRNA126 inhibitor (NI group). HREC cells were treated miR-126 inhibitor (miR-126-inhibitor) (Invitrogen, CA, USA) using Lipofectamine 2000.

### Plasma Analysis

Serum blood glucose, total cholesterol level and HDL were measured (n = 10/group) in the third month after Niaspan treatment using a glucose analyzer (Sannuo, China) and automatic biochemistry analyzer (Beckman Coulter, Inc. Brea, CA). The data are presented as mmol/l values.

### Histologic and Immunohistochemical Analysis

Eyes were removed in the third month after Niaspan treatment and fixed in 4% paraformaldehyde, with phosphate buffered saline (PBS) at pH 7.4 for 2 h at 4 °C, dehydrated with a graded alcohol series, and then embedded in paraffin. The sections were made with a thickness of 5 μm. The sections were then stained with haematoxylin and eosin (HE). For immunohistochemical analysis, sections were prepared from paraffin-embedded tissues and incubated overnight at 4 °C with antibodies to VEGF (1:400, Millipore, Billerica, MA, US); VEGF Receptor 2 (VEGFR2, 1:800, Abcam, Cambridge, UK); Tie-2 (1:50, Santa Cruz Biotechnology, Inc., USA); Claudin-5 (1:100, Invitrogen, USA); Occludin (1:100, Abcam, Cambridge, UK); VCAM-1 (1:500, Abcam, Cambridge, UK); CD45 (1:100, Abcam, Cambridge, UK). Then the sections were stained with biotinylated anti-mouse and anti-rabbit IgG secondary antibody (1:200, Vector Laboratories, Burlingame, CA) for 2 hours followed by horseradish peroxidase streptavidin incubation for 1 hour. Specific labeling was visualized by incubation with diaminobenzidine (DAB, Zhongshan Goldenbridge Biotechnology, China). Finally, the sections were counterstained with hematoxylin (Solarbio Science & Technology, China). Photographs were taken by Leica DMI4000B (Leica Microsystems, Hesse, Germany).

### Western blotting

Western blotting was performed using standard methods. Retinal and HREC proteins were precipitated using RIPA buffer and quantified using a protein assay (Bradford Protein Assay; Bio-Rad, Hercules, CA, US). Equal amounts of protein were separated by electrophoresis on 8–12% dodecyl sulfate-polyacrylamide gel electrophoresis and electroblotted onto polyvinylidene fluoride (PVDF) membranes (Millipore, Billerica, MA, US). The membranes were blocked in 5% skim milk for 2 hour and incubated with antibodies to VEGF(1:1000, Millipore, Billerica, MA, US), VEGFR(1:1000, Cell Signaling Technology, Inc., USA), Ang-1(1:1000, Millipore, Billerica, MA, US), Tie-2(1:200, Santa Cruz Biotechnology, Inc., USA), ZO-1(1:200, Invitrogen, USA), VCAM-1(1:2000, Abcam, Cambridge, UK) overnight at 4 °C. Then the membranes were incubated with secondary antibodies at room temperature for 1 hour. β-actin (1:1000, Zhongshan Goldenbridge Biotechnology, Beijing, China) was used as an internal reference. Finally, the blots were scanned with a ChemiDoc™ MP System (Bio-Rad, CA, US) and the bands were quantified by Image J software.

### Quantitative real-time reverse transcription PCR assays (qRT-PCR)

Total RNA of the retina was isolated using Trizol reagent (Invitrogen, US) and reverse transcribed into cDNA with TransScript First-Strand cDNA Synthesis SuperMix (TransGen Biotech, Beijing, China). The primer sequences for β-actin were (forward, 5′-AGCCATGTACGTAGCCATCC-3′; reverse, 5′- ACCCTCATAGATGGGCACAG-3′); Zo-1 (forward, 5′-ATTTACCCGTCAGCCCTTCT-3′; reverse, 5′-TCGCAAACCCACACTATCTC-3′); Claudin-5 (forward, 5′-ATCGGTGAAGTAGGCACCAA-3′; reverse, 5′-CTGCCCTTTCAGGTTAGCAG-3′); Occludin (forward, 5′-TGAATGGCAAGCGATCATAC-3′; reverse, 5′-TGCCTGAAGTCATCCACACT-3′). The relative amounts of ZO-1, Claudin-5, and Occludin mRNA were detected by qRT-PCR with TransScript Top Green qPCR SuperMix (TransGen Biotech, Beijing, China). All the procedures were performed according to the manufacturer’s instructions. The relative mRNA expression was displayed using 2^−ΔΔCt^ method.

### Quantitative real-time reverse transcription PCR assay for microRNA-126 (miR-126) expression

We performed quantitative real-time reverse transcription-PCR assay for miR-126 expression in retina and HREC. MiRNA fractions were isolated from retina and HREC by using a small RNA isolation kit (Genepharma, China); U6-rRNA was selected as internal controls. PCR reaction used the TaqMan PCR Reagent (Genepharma, China). MiR-126 levels were quantified based on the ratio of microRNA/U6-rRNA using the formula, 2^−ΔΔCt^.

### Measurement of Blood-Retinal Barrier Breakdown Using Evans Blue

Rats (n = 4/group) were sacrificed in the third month, 2% Evans blue dye (Sigma-Aldrich, St Louis, MO, USA) in saline was administered via the tail vein as a blood-retinal barrier (BRB) permeability tracer at 2 h before sacrifice and immediately fixed in 4% paraformaldehyde for 2 hours. Then the anterior segments were removed and the retinas were dissected and spread on glass slides, vitreous side up, and mounted with mounting medium. Pictures were taken by a confocal scanning laser imaging system fitted with krypton-argon lasers (FV1000, Olympus, Japan).

### Quantitative evaluation of Evans blue dye extravasation

Evans blue dye (Sigma-Aldrich, St Louis, MO, USA) was injected through tail vein at a dose of 45 mg/kg. After the dye circulated for 120 minutes, each rat was perfused with PBS (37 °C) to clear the dye. Immediately after perfusion, the eyes were enucleated and the retinas were carefully dissected away. The weight of each retina was measured after thorough drying in a Speed-Vac. Evans blue was extracted by incubating each retina in 0.3 ml of formamide for 18 hours at 70 °C. The extract was filtered through a 30,000 MW filter at a speed of 15000 rpm for 45 minutes at 4 °C. The absorbance of the filtrate was measured with a spectrophotometer at 620 nm and 740 nm, the absorption maximum for Evans blue in formamide. Calculations were based on the external standards dissolved in the same solvent. The concentration of dye in the extracts was calculated from a standard curve of Evans blue in formamide and normalized to the dry retinal weight and the time-averaged concentration of Evans Blue in the plasma.

### Terminal deoxynucleotidyl transferase biotin-dUTP nick end labeling (TUNEL)

Apoptosis was examined by TUNEL assay. TUNEL-positive nuclei GCL of retina were counted. Briefly, after 8 min fixation at room time with ice-acetone solution, cryopreserved tissue sections were incubated with 1 mL of blocking buffer (3% Normal Goat Serum in PBS) for 1 hour. Following incubation, the sections were then in a permeabilization solution (0.1% Triton X-100 in 0.1% sodium citrate) for 2 min on ice. The sections were incubated in 50 μL TUNEL reaction mixture (Roche Diagnostics GmbH, Germany) and incubated for 60 min at 37 °C in the dark. Then sections were counterstained with 4, 6-diamidino-2-phenylindole (DAPI) after the reaction. The sections were then observed under a fluorescence microscope (Olympus, Japan).

### Data and Statistical Analysis

All the data are presented as mean ± SD and analyzed by SPSS 17.0 software. The results were analyzed by ANOVA followed by a least-significance procedure. Differences were considered statistically significant if *P* < 0.05.

## Results

### Niaspan treatment of diabetic retinopathy increases High-Density Lipoprotein and body weight, decreases Serum Total Cholesterol

To test whether Niaspan regulates body weight, blood glucose, serum cholesterol and HDL level, we measured body weight, blood glucose, serum cholesterol and HDL levels in the third month after Niaspan treatment. The Table shows that diabetes significantly increases blood serum glucose, total cholesterol level and decreases body weight, HDL level compared to normal control rats (*P* < 0.05); while Niaspan treatment in diabetic rats significantly decreases serum total cholesterol and increases body weight, HDL level compared to non-treatment diabetic rats (*P* < 0.05) (see the Table 1).

### Niaspan treatment effects on Diabetic Retinopathy

**Figure 1:**
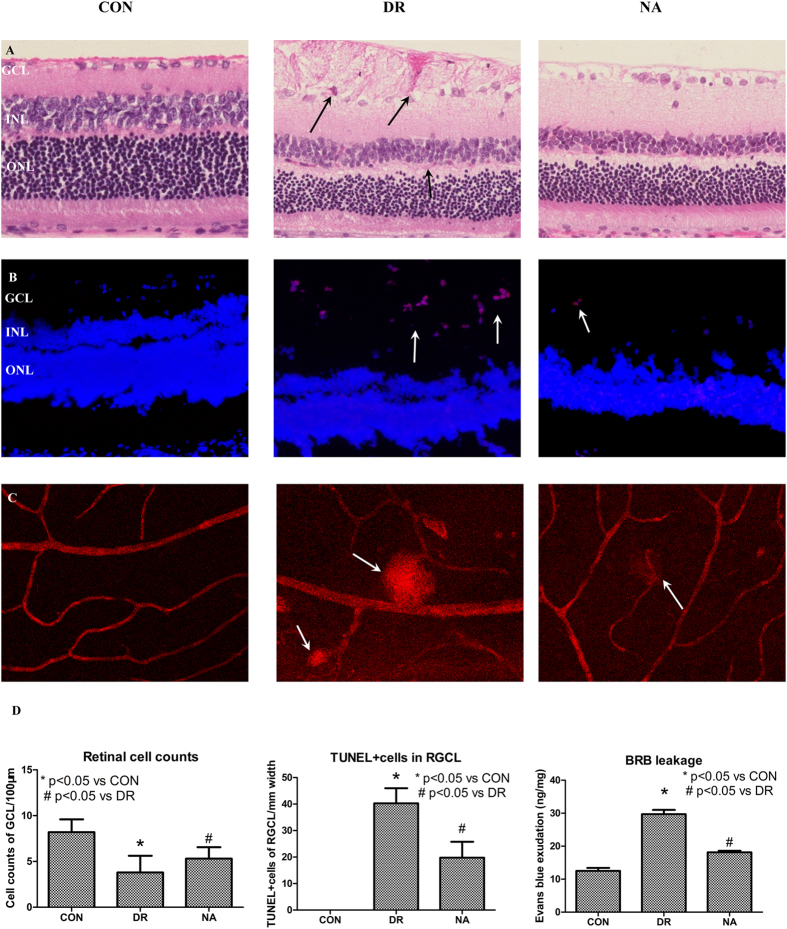
Niaspan treatment of DR rats increases retinal cell counts, and decreases BRB leakage and cell apoptosis. (**A**) HE staining for retina (n = 7/group). (**B**) TUNEL staining of retina (n = 4/group). (**C**) Evans blue assay for BRB leakage (n = 4/group). (**D**) The respective quantitative analysis include of Retinal cell counts of GCL, TUNEL + cells in the RGCL and Evans exudation quantity. *P < 0.05 compared with nondiabetic retina (CON). ^#^*P* < 0.05 compared with diabetic retina (DR).

To test whether DM induces DR and whether Niaspan treatment regulates DR recovery, retinal pathology, cell apoptosis, and BRB leakage were measured. As shown in Fig. 1: 1) Non-diabetic animal showed a normal retina, all cell layers of retina were clear and neatly arranged. In the DR group, cells were disorganized after DM modeling. Obvious vacuolated, hemorrhage, inflammatory cell infiltration in GCL were observed (Fig. 1A). Moreover, there are significantly reduced cell number of retinal ganglion cell layer (RGCL) and increased the numbers of TUNEL (+) cells in the GCL of the diabetic retina (*P* < 0.05) compared with to CON retina (Fig. 1A,B and D). The breakdown of the BRB induced by diabetes was assessed by Evans blue extravasation from retinal vessels. As a first approach, Evans blue was shown to be confined to the retinal blood vessels without any leakage occurring in control retina. However, the dye was shown to leak from the vessels to the surrounding tissue in diabetic rats (Fig. 1C). The quantitative measure of Evans blue dye, from the retinal tissue, confirmed the data obtained by fluorescence microscopy. Diabetes increased the BRB permeability in diabetic rats (*P* < 0.05) compared to control rats (Fig. 1D). 2) The Niaspan-treated retina significantly attenuated the retina edema, hemorrhage and ganglionic layer was neatly arranged (Fig. 1A). Meantime, treatment with Niaspan was able to significantly prevent the reduction of retinal cells and decrease retinal cell apoptosis (*P* < 0.05) compared with that of the diabetic retina (Fig. 1A,B and D). Furthermore, the Niaspan treatment also significantly prevented BRB breakdown in diabetic rats when compared to untreated diabetic animals (Fig. 1C,D).

### Niaspan prevents the alterations in the distribution of Tight Junction proteins in retinal vessels induced by Diabetes

**Figure 2:**
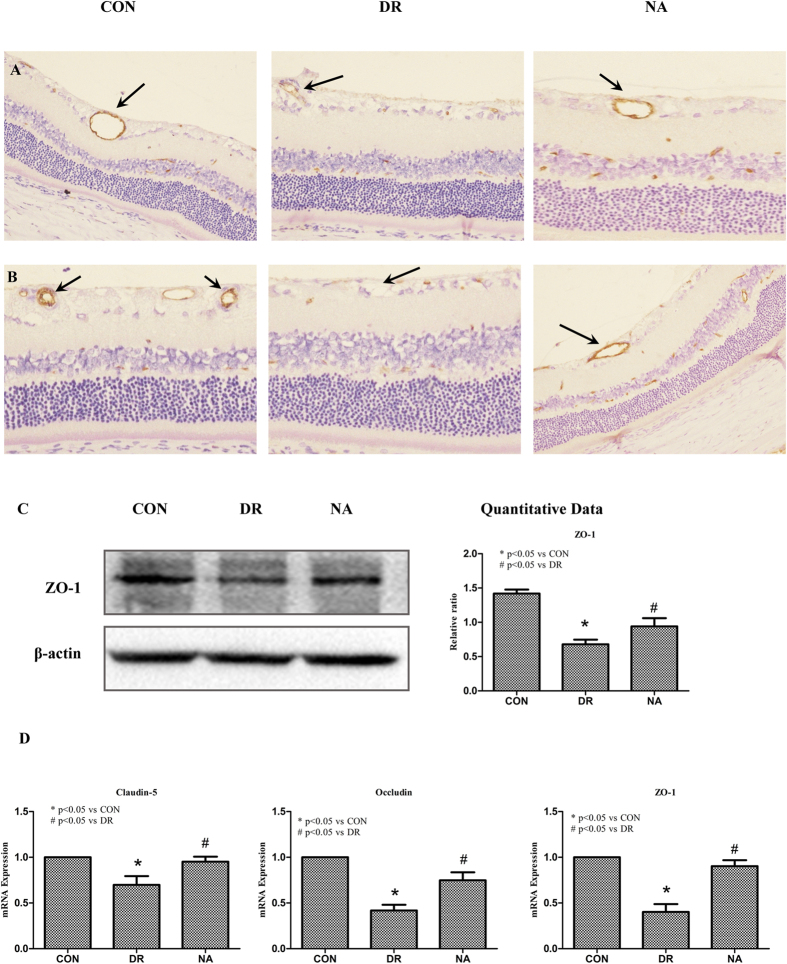
Niaspan treatment increase tight junction protein expression. (**A**) The tight junction protein Claudin-5 immunostaining. (**B**) The tight junction protein Occludin immunostaining. (**C**) Western blot analysis to determine ZO-1 protein expression in the third month control retina, diabetic retina, and the retina of Niaspan-treated rats. Lane loading was normalized by reblotting with a monoclonal anti-β-actin antibody. Relative expression levels of ZO-1 (n = 4/group) are present. (**E**) RT-PCR experiments to determine Claudin-5, Occludin, ZO-1 expression in the retina. *P < 0.05 compared with nondiabetic retina (CON). ^#^*P* < 0.05 compared with diabetic retina (DR).

In order to establish a correlation between the effects observed on the BRB permeability and tight junction organization. Claudin-5, Occludin, and zonula occludens (ZO-1), tight junction proteins, immunohistochemistry staining and Western blot assay were employed. The pictures revealed that there was a significant decrease in Claudin-5 and Occludin expressions in the vessel of diabetic retina when compared to control rats (Fig. 2A,B). DR also significantly decreases ZO-1 expression in the diabetic retina when compared to control retina (*P* < 0.05, Fig. 2C). Treatment with Niaspan significantly increases Claudin-5 and Occludin expression in the vessel of retina when compared to non-treatment diabetic rats (Fig. 2A,B). Western blot analysis shows that Niaspan treatment significantly increased the expression of ZO-1 when compared to non-treatment diabetic rats (*P* < 0.05, Fig. 2C).

RT-PCR also shows there was a significant decrease in Claudin-5, Occludin and ZO-1 in diabetic rats (*P* < 0.05) when compared to control rats. Treatment with Niaspan was able to significantly prevent the decrease in Claudin-5, Occludin and ZO-1 (*P* < 0.05) when compared to diabetic rats (Fig. 2E).

### Niaspan ameliorates the decrease of miR-126 and regulates miR-126 target gene expression in Diabetic Retinopathy

**Figure 3:**
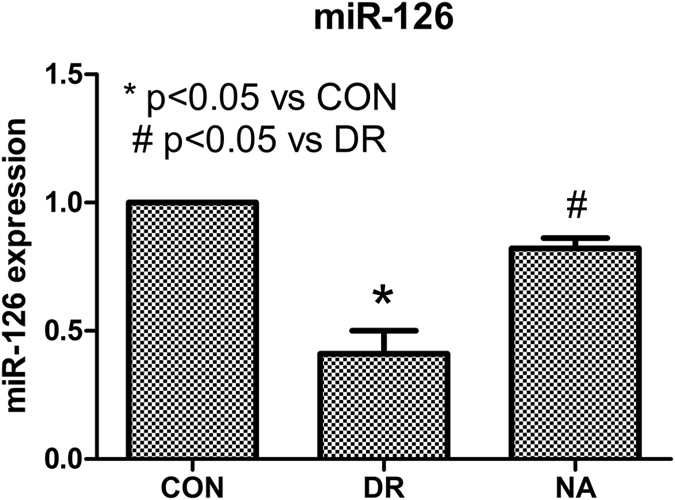
Expression of miR-126 in the retina of diabetic retinopathy. Real time PCR was performed for miR-126 expression. The reduction of miR-126 levels was detected in the retina of diabetic rats. Niaspan treatment increase retina miR-126 expression. *P < 0.05 compared with nondiabetic retina (CON). ^#^*P* < 0.05 compared with diabetic retina (DR).

MiR-126, the miRNA considered to be specially expressed in endothelial cells, is strongly associated with angiogenesis. To understand the mechanisms of Niaspan treatment induced protective effect in DR rats, miR-126 expression was measured. Our study shows that the reduction of miR-126 levels was detected in the retina of diabetic rats (*P* < 0.05) when compared to control group. However, the Niaspan treatment significantly enhance the level of retinal miR-126 expression (*P* < 0.05, Fig. 3).

### Niaspan treatment of Diabetic Retinopathy decreases miR-126 target gene VEGF/VEGFR and VCAM-1 expression in the retina

**Figure 4:**
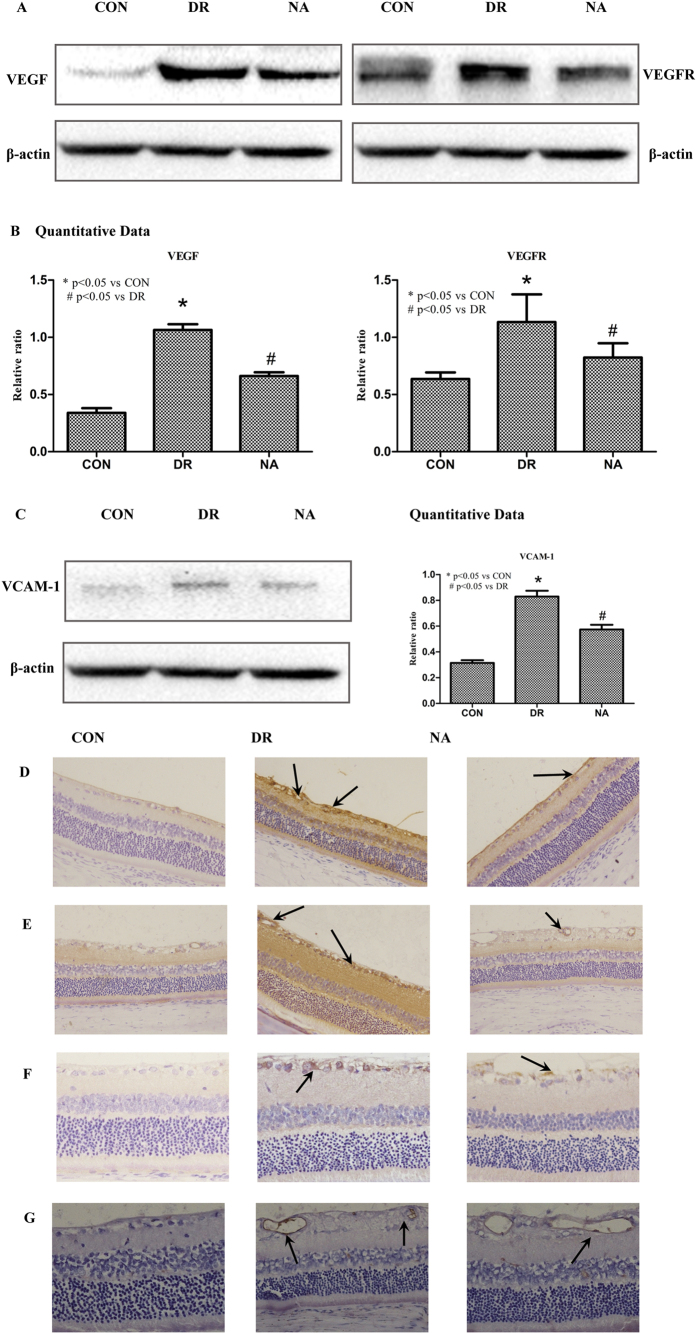
Niaspan treatment decreases VEGF/VEGFR and VCAM-1/CD45 in the DR rats compared to non-Niaspan treatment DR rats. (**A,B**) Western blot and quantitative measurement for VEGF and VEGFR expression in the retina (n = 4/group). (**C**) Western blot and quantitative measurement for VCAM-1 expression in the retina (n = 4/group). (**D**) Immunostaining for VEGF expression in the retina (n = 7/group). (**E**) Immunostaining for VEGFR expression in the retina (n = 7/group). (**F**) Immunostaining for and VCAM-1 expression in the retina (n = 7/group). (**G**) Immunostaining for CD45 expression in the retina (n = 7/group). *P < 0.05 compared with nondiabetic retina (CON). ^#^*P* < 0.05 compared with diabetic retina (DR).

MiR-126 has several target genes such as VEGF and VCAM-1^17^,^18^. To test whether Niaspan treatment regulates miR-126 and therefore regulates target gene VEGF and VCAM-1 expression. VEGF/VEGFR2 and VCAM-1/CD45 expressions were measured. As shown in Fig. 4: 1) Western blotting shows that DR significant increases VEGF/VEGFR and VCAM1 expression in retina in diabetic rats (*P* < 0.05) when compared to control rats. Treatment with Niaspan significantly prevents the increased in VEGF/VEGFR as well as VCAM-1 expression (*P* < 0.05) in the DR retina when compared to non-treatment diabetic rats (Fig. 4A,B and C). 2) Immunohistochemistry shows that there was a significant increase VEGF/VEGFR and VCAM-1/CD45 in diabetic rats when compared to normal control rats. Treatment with Niaspan was able to significantly prevent the increase in VEGF/VEGFR and VCAM-1/CD45 when compared to diabetic rats (Fig. 4D–G).

### Niaspan treatment of Diabetic Retinopathy increases miR-126 target gene Ang-1/Tie-2 expression in the retina

**Figure 5:**
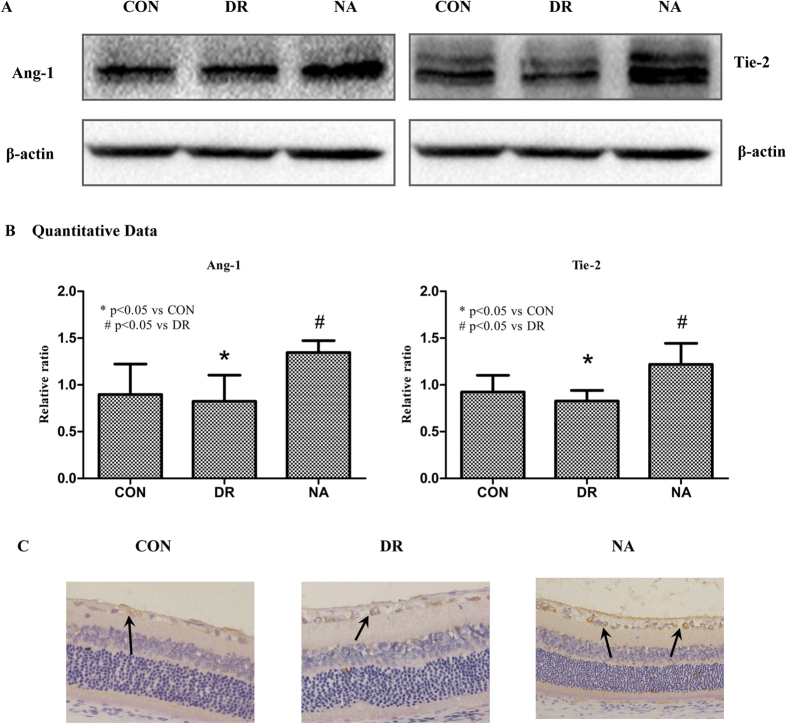
Niaspan treatment increases Ang-1 and Tie-2 in the DR rats compared to non-Niaspan treatment DR rats. (**A,B**) Western blot and quantitative measurement for Ang-1 and Tie-2 expressions in the retina (n = 4/group). (**C**) Immunostaining and quantitative data for Tie-2 expression in the retina (n = 7/group). *P < 0.05 compared with nondiabetic retina (CON). ^#^*P* < 0.05 compared with diabetic retina (DR).

Ang-1 is important angiogenic factors. Tyrosine kinase with Ig and EGF homology domains-2 (Tie-2) is a transmembrane tyrosine kinase receptor and is expressed by both vascular and lymphatic endothelial cells, and it promotes angiogenesis and vascular stabilization^19^. To test the mechanisms underlying Niaspan induced vascular remodeling, we measured Ang-1 and Tie-2 expressions in the retina. Western blotting shows that Ang-1 and Tie-2 level have not significantly difference between normal control group and diabetic group (*P* > 0.05). However, treatment with Niaspan significantly increase in Ang-1 and Tie-2 (*P* < 0.05) expression when compared to non-treatment diabetic rats (Fig. 5A,B). Meantime, Immunohistochemistry consistent with Western blot assay shows that there is no significant difference in Tie-2 between CON group and DR group; while Niaspan treatment significantly increases Tie-2 expression in the retina compared with non-treatment diabetic retina (Fig. 5C).

### Niaspan decreases VEGF and increases Ang-1 in the Human Retinal Endothelial Cells

**Figure 6:**
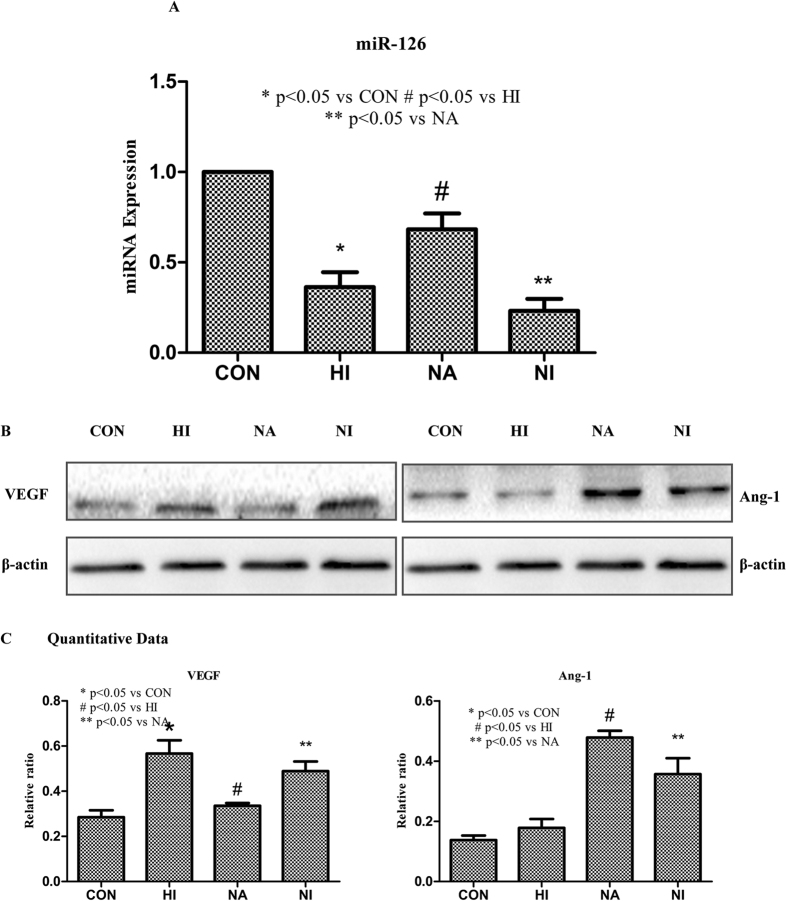
Niaspan treatment decreases VEGF and increases Ang-1 expression in the HREC compared to non-Niaspan treatment HREC. (**A**) RT-PCR experiments to determine miR-126 expression in the HREC (n = 3/group). (**B,C**) Western blot and quantitative measurement for VEGF and Ang-1 expressions in the HREC (n = 4/group). *P < 0.05 compared with nondiabetic HREC (CON). ^#^*P* < 0.05 compared with hyperglycemia treatment (DR). **P < 0.05 compared with the HREC without miR-126 inhibitor pretreatment.

To further investigate the relation of miR-126 and Niaspan, this experiment was performed in the vitro. As shown in the Fig. 6: hyperglycemia significantly decreases miR-126 expression in the hyperglycemia-induced cells and miR-126-inhibitor pretreatment cells compared to the normal control cells (*P* < 0.05). However, Niaspan intervention markedly increases the miR-126 expression in the HREC (*P* < 0.05, Fig. 6A).

We examined the effect of miR-126 on VEGF and Ang-1 expressions in HREC pretreated with miR-126-inhibitors by immunoblotting. Western blot shows that hyperglycemia-induced cells increases the expression of VEGF compared to the CON (*P* < 0.05). However, treatment with Niaspan decreases VEGF expression and increases Ang-1 expression (*P* < 0.05). Pretreatment with miR-126-inhibitors bring down the reduction of VEGF and the increase of Ang-1 expression which resulted in by Niaspan in hyperglycemia-induced HREC cells (*P* < 0.05, Fig. 6B,C).

## Discussion

In this study, by a common animal model of STZ-induced DR, we show that STZ injection induced diabetes significantly induces DR, while long term of Niaspan treatment diabetes rats slowed down the formation and development of DR, by increasing serum HDL and retinal miR-126 expression as well as increasing Ang1/Tie2 expression. Moreover Niaspan treatment of DR also decreases miR-126 target gene VEGF/VEGFR and VCAM-1/CD45 expressions in retina of DR rats.

HDL, in contrast to low-density lipoprotein (LDL), acts as an anti-atherogenic lipoprotein by promoting of macrophage cholesterol efflux and protecting directly endothelial effects. HDL also stimulates production of endothelial nitric oxide and induces anti-apoptotic, anti-inflammatory and anti-thrombotic properties^20^. Clinical epidemiological studies have demonstrated that dyslipidemia/dyslipoproteinemia is significantly correlated with DR patients^21^. Serum Apo-B and Apo-B/Apo-A ratio were the most significant metabolic risk factors for PDR^22^. Decreasing HDL/LDL ratio is correlated with more severe retinopathy^23^. Others have previously described, during the course of diabetes, injury and apoptosis occur early in endothelial cells and retinal ganglion cells^24^. Endothelial and pericytes cell loss is one of the earliest and key manifestations of diabetic retinopathy, it leads to BRB breakdown^25^. Our study shows that Niaspan treatment of diabetes rats significantly prevents and reverses these changes by increasing serum HDL level and decreasing hemorrhage, leukocyte infiltration and apoptosis in GCL of diabetic retina. Niaspan treatment also reduces endothelial cell injury and BRB.

Diabetic retinopathy, is characterized as a microvascular complication (leukocyte adhesion, apoptosis of vascular (endothelial cells and pericytes) of involves multiple mediators such as pro-inflammatory cytokines, chemokines and adhesion molecule^26^. The changes progress to involve breakdown of the BRB causing diabetic macular edema, the most leading cause of vision loss in DR^27^. Diabetes causes hypoxia and ischemia of the retina and triggers a series of structural and functional alterations in the retinal endothelial cells^28^. At the same time, Claudin-5, Occludin, and ZO-1 are the main proteins involved in the tight junction complex, which is essential for the control of endothelial permeability^29^. Retinal endothelial barrier integrity is also implicated in the maintenance of vascular permeability and the development of DR^30^. Our results showed Niaspan-treated DR rats significantly prevent the decreased expression of tight junction proteins in the retina. As a consequence, the increased tight junction protein expression by Niaspan treatment may contribute to improve the integrity of BRB.

Circulating miR-126 expression was significantly decreased in type 1 diabetes patients than that of the healthy controls^31^. The expression of miR-126 was reduced in the retina tissue of streptozotocin-induced diabetic rats^32^. Diabetes also decreases circulating endothelial progenitor cells (EPC) miR-126 expression and impair EPC function via its target, Spred-1 and VEGF^33^. Decreasing miR-126 level induces endothelial injury and participate in the development and progression of diabetic vascular complications^34^. Consistent with previous studies, we found that DR significantly decreases retina miR-126 expression compared to normal control rats. However, Niaspan-treated DR significantly increased the expression of microRNA-126 which has been proven is a key positive regulator of angiogenic signaling in endothelial cells and of vascular integrity^18^.

MiR-126 has many target genes, such as VEGF and VCAM-1. Upregulation of VEGF-responsive miRNA constituted key miRNA signatures, reflecting ongoing pathologic changes of early DR^35^. MiR-126 may halt the hypoxia-induce neovascularization by suspending the cell cycle progression and inhibiting the expression of VEGF^32^. At the same time, DM-induced DR significantly increases retina VEGF and inflammatory factors expression^36^. VEGF has a requisite role in early endothelial cell differentiation, proliferation, vessel formation and the breakdown of BRB. So, the decreased expression of VEGF may result in reduction of BRB disruption and inhibiting of neovascularization. Here we show that Niaspan-treatment significantly increases retina miR-126, but decreases retina VEGF/VEGFR2 expression. Moreover, *in vitro* experiment suggests that the inhibition of miR-126 may influence the effects of Niaspan on VEGF. So, Niaspan could suppress the high levels of VEGF and finally overcome the diabetic-induced retinal neovascularization by increasing miR-126.

VCAM-1 is an adhesion molecule that mediates leukocyte attach to the vessel wall, transmigrate through the endothelium and move to the infected or injured tissue^37^. Leukocyte adhesion to the retinal vasculature is one of the earliest pathological changes observed in the development of diabetic retinopathy and leads to enhanced vascular permeability, endothelial cell damage and capillary non-perfusion^38^, which is considered to play an important role in the development of DR^39^. Increasing VCAM-1 expression and leukocyte infiltration may induce disorganization of junction proteins in the retinal endothelium may lead to vascular leakage and breakdown of the BRB^26^. We found that diabetes significantly increases VCAM-1 and CD45-positive leukocyte infiltration into the retina compared to normal control rats’ retina. Niaspan markedly decreased VCAM-1 expression and CD45-positive leukocytes infiltration in the retina. A report suggests that endothelial cells express miR-126, which inhibits VCAM-1 expression^17^. Therefore, decreasing inflammation by Niaspan treatment may play important role in Niaspan induced decreasing BRB and DR. Niaspan inhibits leukocyte adhesion and inflammatory may via regulating VCAM-1 expression by miR-126.

Previous studies also shown that miR-126 regulates Ang-1 signaling and vessel maturation by targeting p85 β^40^. The angiopoietins (Angs) are a family of growth factors that bind to the endothelial receptor Tie-2 and regulate vascular stabilization and function^41^. Ang-1 prevents vascular endothelial cell injury in the diabetic retina^42^ and promotes survival of endothelial cells without causing endothelial cell proliferation and stabilizes tubular networks. In another word, that increased expression of Ang-1 in the retina does not cause an increase in the number of retinal vessels^43^. Ang-1 is also a critical maintenance factor that stabilizes blood vessels and decreased mild breakdown of the blood-retinal barrier. Moreover, Ang-1 seems to function later in vascular development, contributes to the stabilization and maturation of growing blood vessels^44^. Targeted regulation of Ang-1 may offer a therapeutic approach as an adjunct treatment for the microvascular complications associated with DR^45^. In this study, we found that Niaspan treatment of DR significantly increases miR-126 and Ang1/Tie2 signaling activity in the retina. Meantime, inhibition of miR-126 weakens the increase of Ang-1 expression induced by Niaspan in the HREC. Hence, Niaspan regulates Ang1/Tie2 expression may get through miR-126 pathway.

**Figure 7:**
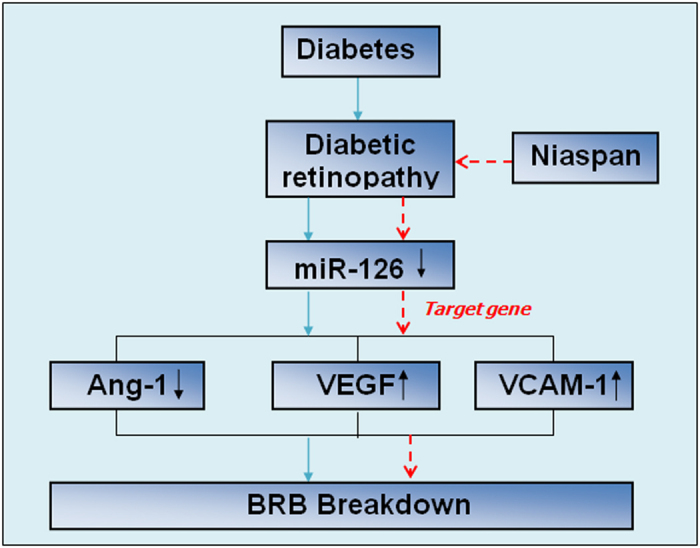
Role of miR-126 in diabetic retinopathy. BRB: Blood–retinal barrier. Blue arrow indicates promotes the following indicators. Red short dash line arrow indicates inhibits the following indicators.

In conclusion, our data suggests that the Niaspan treatment of DR increases of retina miR-126, Ang-1/Tie-2 level, decreases VEGF/VEGFR and VCAM-1 expressions, which may contribute to the stabilization and maturation of growing blood vessels, decrease BRB damage and neovascularization thus slow down the progression of DR. The miR-126 may play an important role in the regulation of DR procedure. All the procedure have shown in the picture Fig. 7. Further investigation of mechanism of miR-126 would be important in developing new therapeutic targets for preventing and reversing diabetic retinopathy.

## Additional Information

**How to cite this article**: Wang, Y. and Yan, H. MicroRNA-126 contributes to Niaspan treatment induced vascular restoration after diabetic retinopathy. *Sci. Rep.* **6**, 26909; doi: 10.1038/srep26909 (2016).

## Figures and Tables

**Table 1:** Body weight, blood glucose, serum total cholesterol level, and HDL were determined after 3 month of diabetes in the presence or absence of Niaspan treatment in STZ-induced rats.

Group	CON	DR	NA
Body weight	357.10 ± 32.54	259.50 ± 16.64*	321.80 ± 24.98^#^
Blood glucose	5.73 ± 0.91	29.39 ± 2.85*	28.35 ± 2.79
Total cholesterol	1.56 ± 0.36	2.64 ± 0.35*	1.97 ± 0.40^#^
HDL	1.15 ± 0.17	0.79 ± 0.15*	1.02 ± 0.23^#^

All results are expressed as mean ± SD. n = 10/group. *P < 0.05 compared with nondiabetic rats. ^#^*P* < 0.05 compared with diabetic rats.
